# Capturing the Temporal Sequence of Interaction in Young Siblings

**DOI:** 10.1371/journal.pone.0126353

**Published:** 2015-05-21

**Authors:** Michal Perlman, Mark Lyons-Amos, George Leckie, Fiona Steele, Jennifer Jenkins

**Affiliations:** 1 Department of Human Development and Applied Psychology, University of Toronto, Toronto, Canada; 2 Centre for Multilevel Modeling, Graduate School of Education, University of Bristol, Bristol, United Kingdom; Purdue University, UNITED STATES

## Abstract

We explored whether young children exhibit subtypes of behavioral sequences during sibling interaction. Ten-minute, free-play observations of over 300 sibling dyads were coded for positivity, negativity and disengagement. The data were analyzed using growth mixture modeling (GMM). Younger (18-month-old) children’s temporal behavioral sequences showed a *harmonious* (53%) and a *casual* (47%) class. Older (approximately four-year-old) children’s behavior was more differentiated revealing a *harmonious* (25%), a *deteriorating* (31%), a *recovery* (22%) and a *casual* (22%) class. A more positive maternal affective climate was associated with more positive patterns. Siblings’ sequential behavioral patterns tended to be complementary rather than reciprocal in nature. The study illustrates a novel use of GMM and makes a theoretical contribution by showing that young children exhibit distinct types of temporal behavioral sequences that are related to parenting processes.

## Introduction

Young siblings interact with one another frequently and their interactions have been linked to children’s social cognition, learning, friendship quality and well-being [1]. Sibling relationships serve as a training ground for children, shaping the nature of their social exchanges [2], [3], [1]. Research has identified aggregate processes in the sibling relationship (e.g., overall negativity or positivity) that are important in predicting later relationship functioning and child well-being. However, we have little information about moment-to-moment processes in sibling interaction. The first goal of this study was to use a methodology, new to sibling research, to identify common sequential patterns during a 10-minute interaction between 18-month-old children and their older siblings (mean age of 4.5 years). Second, we sought to determine whether sequential patterns were associated with characteristics of the child or family. Third, we examined the extent to which sequences of interaction between siblings are reciprocal or complementary.

### Methodologies for the study of sequential behavior in sibling interaction

Social exchanges are inherently sequential processes that evolve over time [4]. Yet, much of the research on children’s interactions has focused on averages of different types of behaviors (e.g., aggression, prosocial behavior). Averages obscure differences between individuals in the *course* of interaction. For example, by examining average aggression in an interaction researchers equate children who begin interactions with low levels of aggression but then escalate their use of aggression with children who are moderately aggressive throughout. Yet, the effect of these two patterns on partners may be quite different. Thus, we need to explore methods that allow us to move beyond simply averaging behaviors across time.

The most common ways to look at sequences in interaction have been conditional probabilities in the form of sequential analysis [4], [5], [6]. The conditional probability approach answers the question: what is the likelihood of specified behaviors following a target behavior? For example, Perlman and Ross [4] looked at the likelihood that a power assertive move by one sibling would be followed by either reciprocal or complimentary behaviors by the other sibling. Two consequences of this methodology need to be considered. First, the approach requires many instances of the target event and the interactional partner’s behavior on the next move (If-then sequences). These if-then sequences are drawn from the whole period of the interaction. Thus, in order to have enough instances of the power assertive move Perlman and Ross equated power assertive moves at the beginning, middle and end of the interaction. It is possible that such moves have different meaning at different points during the interaction. This is suggested by Ross, Ross, Stein & Trabasso’s [7] study of conflict discussions in 4–12 years old siblings. They found that *first offers* that met both children’s goals were associated with good conflict outcome: thus where a move came in the sequence of interaction affected the outcome. Second, sequential analysis requires specification of the lag between behaviors under scrutiny. Often lag 1 is chosen as the target behavior and the other person’s behavior that immediately follows it (i.e., X + 1). While this is a reasonable starting point it assumes that the impact of a target behavior will be immediate. Other researchers have made a different set of untested assumptions by choosing lags of different durations (See [8] for a discussion of studying different interaction lag times).

In this paper we present growth mixture modeling (GMM) as an alternative methodology to sequential analysis, but one that is sensitive to the sequential nature of unfolding interactions. This technique allows us to examine whether there are patterns in the moment-to-moment interactions of siblings, observed over a ten-minute period, that characterize different groups of children. For instance, there may be a group of children who start interactions off positively, but quickly lose interest and disengage from their partner. This might contrast with a group of children who are positive throughout. The GMM combines the strengths of individual growth curve analysis with latent class analysis. Individual growth curve analysis allows us to model each person’s starting point and change over time in behavior [9]. Using latent class analysis, change in behavior over time is also examined, but the technique looks for the smallest number of classes or groupings that summarize the patterns of sequenced behavior [10]. By combining across these methods, GMM provides descriptions of classes of behavior but also allows for within-class variation in individuals’ starting points and growth rates [11], [12], [13]. In effect it provides us with a means for identifying typologies in sequences of individual child behavior. While recognizing the value of this approach to sequential data analysis, there are limitations related to the number of features that can be simultaneously analyzed (see discussion). For this reason younger and older children’s trajectories are analyzed separately.

### Typologies of sibling interaction

Typologies of sibling interaction have been previously identified but never empirically extracted using moment-by-moment observational data. These provide interesting, person-oriented analyses that capture the multidimensional nature of interactions for siblings across the life span. Typically they characterize relationships along the dimensions of positivity, negativity and engagement [14–19]. For instance, using cluster analysis Brody, Stoneman and McCoy [20] found three typologies when children were between the ages of 7 and 12-years (harmonious, conflicted and typical which was characterized by both warmth and conflict) and two when children were 11–16 years-old (harmonious and moderately conflicted). Other researchers have found similar categories that generally include a positive/harmonious group, a negative group and an indifferent or casual group that reflects little engagement between the siblings [17]. A mixture model analytic approach, representing a simpler version of the analytic approach used in the current study, has been shown to be more sensitive than other typology analytics to subtle differences in how siblings interact [19].To our knowledge, all the typology studies that have been carried out on siblings, have been based on across-time averages [20]. Yet, as discussed above, relationship processes unfold over time with the sequence of behaviors having implications for the outcome [7]. Our first goal was to determine whether during a 10-minute observed interaction, temporal typologies of interaction could be identified for both younger and older siblings.

We expected to see a harmonious, largely unchanging temporal sequence, without an increase in negativity or disengagement, for a substantial proportion of both younger and older children. This is based on past findings of a harmonious typology [14]. Past typology research has identified a somewhat negative sibling interaction style for some children [14–19]. However, since that research was based on aggregate data, we speculate that this group could be made up of different subgroups of children including one group of children whose interaction deteriorates and a second group who are able to recover from deterioration in their interactions. Finally, based on past finding of an “indifferent” or “casual” sibling interaction style, we expected a group of children who would disengage over time. Once temporal typologies of interaction have been identified we can explore whether these are associated with expected predictors.

#### The role of maternal mind-oriented parenting and positivity in predicting sibling typologies

Several different types of maternal behavior predict the quality of sibling interactions [21], [22]. Maternal positivity, defined as warmth and engagement, has been found to relate to sibling relationship quality [23], [22]. Observational learning has been offered as a potential mechanism to explain this association [23].

There are several reasons to think that mind-oriented parenting will also explain sibling relationship quality. We include both sensitive parenting and reflective parenting within this construct as they have been shown to relate to one another [24]. First, an intervention study in which mothers were taught to help siblings understand the motivations and goals of their sibling resulted in less negativity, more compromise and more perspective taking towards the sibling [25]. Second, mind-oriented parenting has been shown to be associated with higher levels of social understanding in children [24], [26]and social understanding has in turn been found to be associated with more positive sibling relationships [27]. Both Bowlby [28], with the goal-corrected partnership, and Kochanska, Aksan, & Carlson [29] with the mutually rewarding orientation, have argued that cognitive/affective structures develop in the preschool period that foster the child’s ability to interact successfully with others. These structures are influenced in part, by maternal behavior that is oriented and sensitive to the feelings and goals of the developing child [29]. We expected mind-oriented parenting and maternal positivity to be associated with the most positive typologies of temporal behavioral sequences: those in which siblings would be able to engage in a positive and sustained way that does not deteriorate and where recovery from altercations would be evident.

#### The role of child characteristics in predicting sibling typologies

First, emotion regulation skills (including delay of gratification, ability to consider the consequences of one’s actions and to manage attention effectively) play a role in enabling children to minimize disruption to the quality of their interactions [30], [31] and recover from minor conflicts. These skills develop over the preschool period, making it important to take child age into account.

Second, birth order influences on typologies were expected. Older siblings experience more power in the sibling relationship because of their greater competence, the hierarchical nature of families [32] and social norms that encourage younger children to follow the directions of older children [33]. High interpersonal power is associated with open displays of negativity, whereas low power is associated with disengagement [34]. Consequently, we expected a pattern of negativity and disengagement for older children. For younger children we expected this pattern to involve only disengagement. Furthermore, based on the development of emotion regulation we expected to see older, but not younger children, recover from a period of negativity or disengagement by reengaging positively with their sibling.

Finally, gender, age-gap and SES were included as covariates in the analyses because they have been reported to relate to the quality of sibling interaction. Because these relationships have been weak and inconsistent [24], [32] no specific hypotheses were made with respect to these covariates.

### Reciprocity or complementarity in sibling interaction

Sibling interactions have been described as reciprocal (i.e. egalitarian) or complementary (i.e. hierarchical) [35]. This refers to the context of interaction (e.g. the reciprocity of joint play versus the complementarity of an older child teaching a younger one) but also to the direct response that a child makes to a sibling’s behavior. For instance, within a sequential analysis framework the most common response to a sibling’s behavior is to respond ‘in kind’ [36], [37] a negative response to negative initiation and a positive response to a positive initiation. The same ‘in-kind’ is also true based on aggregate measures: when one child expresses positivity towards a sibling, the feelings are reciprocated by the sibling [38], [21]. These results, however, based either on two-step exchanges or aggregated measurement may obscure the conclusion to date that sibling interactions are largely reciprocal or ‘tit-for-tat’. As previously described, averaging across an episode and brief sequential approaches are not sensitive to the temporality of the data. Based on the studies described above we expected some sibling pairs to show the same typology of response, but given differences in developmental competencies between older and younger siblings we also expected to see mismatches in the types of temporal sequences they exhibited.

### Hypotheses

1. We expected to find evidence for discrete typologies of temporal sequences of child behavior for younger and older children, based on sequences of positivity, negativity and disengagement. Specifically we expected to find a:
  - **Harmonious class** for both of our age groups. We expected that children in this class would exhibit stable or rising positivity and stable or falling negativity and disengagement.
  - **Deteriorating class** of children who exhibit increases in negativity and declines in positivity over time. Given the power differential between siblings we expected to see this pattern for older, but not younger siblings.
  - **Recovery class** of children consisting of initial deterioration in the interaction (increases in negativity or disengagement) followed by a recovery (a move towards greater positivity subsequent to deterioration). Due to the high emotion-regulation demands of this pattern, we expected this class for the older siblings only.
  - **Casual class** of children who disengaged from their siblings over the course of interaction. We expected to see this class for older and younger siblings, however, due to the power differential we expected this pattern to involve lower negativity and higher positivity for younger than it would for older siblings.
2. We expected that after controlling for the covariates described above (e.g., age and gender) mothers of *harmonious* and *recovery* children will show more mind-oriented parenting (i.e., maternal sensitivity and reflective capacity) and positivity than mothers of children in the deteriorating/disengaging interaction group.
3. Based on findings that children tend to reciprocate behaviors we expected to see overlap between some siblings on older and younger typologies. We also expected to see mismatches, however, because of the developmental differences between older and younger siblings.

## Methods

### Sample

The sibling data described in the current paper was part of a longitudinal sibling study (Kids, Families, Places), which investigates genetic and environmental influences on young children’s development. Institutional Review Boards (IRB) at the University of Toronto and McMaster University approved all protocols. Written informed consent was obtained from the guardians of all participants in keeping with the IRB’s guidelines. All of the women giving birth to infants in the cities of Toronto and Hamilton between February 2006 and February 2008 were considered for participation. Families were recruited through a program called *Healthy Babies Healthy Children*, run by Toronto and Hamilton Public Health Units, which contacts the parents of all newborn babies within several days of the newborn’s birth. Inclusion criteria for participating in this study included an English-speaking mother, a newborn > 1500 grams and at least one older child < 4 years. In Toronto 34% of the families we approached agreed to take part. At Time 1 (infants were 2 months old), 501 families, recruited between 2006 and 2008, took part in the study. These families were followed up when ingsing was around 18 months old. The older sibling could be up to 5.5 years old (mean = 4.05). Families were a mix of 2-child families (N = 259), 3-child families (N = 59) and 4-or-more-child families (N = 18). Only two children per family were included for observational tasks (sibling 1 = newborn at Time 1, Sibling 2 = next in age older sibling < 4 years old at Time 1) because of burden on families and cost considerations. The mean age difference between the younger and older siblings was 2.45 years. Six hundred and seventy two children from 397 families took part at Time 2. The visit was ended prior to completion of the sibling interaction task for 61 families (see discussion below). Thus, for the purposes of this analysis we had data from 336 families. There were 345 boys and 327 girls. Gender composition of the sibship was coded as all-boy (N = 85), all-girl (N = 76), boy-girl mixed-gender sibships (N = 90) and girl-boy mixed-gender sibships (N = 85).

Our sample had an average of 4.53 (SD = 1.01) people living in the household and an average maternal personal income that fell between 30,000–39,999 which is similar to 2006 Canadian census data. Compared to the general population, our sample had a somewhat higher proportion of Canadian born (57.4% vs. 47.6%) and better educated (54.5% vs. 30.6% earned a bachelor degree or higher) mothers. To some extent, these differences reflect our language criteria and they also reflect an inherent issue in longitudinal studies which is that parents who participate tend to be more educated [39].

### Procedure

Two female data collectors spent an average of two hours in each participating family’s home. Children were observed interacting with their mothers and each other and direct testing of socio-cognitive and cognitive skills were carried out using standard measures. Mothers also completed paper and pencil measurements for a maximum of four children per family. Interviewers were trained extensively before they began data collection. The sibling interaction was conducted at the end of the visit. Interviewers were instructed to be sensitive to the needs of families and to omit the sibling interaction component if they sensed that they were intruding on the family’s schedule or children were tired. Data collection was potentially more intrusive when families had fewer rooms and other resources. This was tested and results are presented below. They suggest that interviewers were respectful of family resources as families with fewer rooms completed the sibling interaction observation less often than families with more rooms. SES status, including the number of rooms in the home, was included as a covariate to mitigate the potentially biasing effects of differences between completers and non-completers. One interviewer was present in the room but attended to administrative work. All video data were coded as described below.

### Measures

A list of the various measures we used is provided below.

#### Sibling interaction

Interviewers provided pairs of siblings with toys chosen to elicit pretend play and asked the children to play together for 10 minutes while being videotaped. Behaviors were coded in 20-second snapshots consistent with work by Volling, McElwain & Miller [40] who used brief snapshots in coding family interactions and researchers who have used this methodology to capture interactions in other settings [41]. Thus, a ten-minute observation yielded a total of 30 snapshots (3 per minute X 10 minutes). After observing each 20-second snapshot, coders coded older and younger siblings’ behaviors separately. Initial coding was conducted on micro codes that captured each child’s behavior within the categories of negativity and positivity. A snapshot was identified as “disengagement” when neither positivity nor negativity were noted. Because the temporal sequences of all codes would have been overwhelming for the analysis and many of the codes were low frequency, only the aggregate codes of negativity and positivity were used. Positivity included the following behaviors: play (which consisted of simple, pretend and cooperative forms of play); positive response to a move initiated by the sibling and positive emotion (which consisted of smiling, laughing and singing). Negativity included the following behaviors: physical aggression; verbal aggression; property disputes; resisting the sibling; negative response to a move initiated by the sibling and negative emotion (which consisted of crying and screaming). Codes could co-occur within the 20-second snapshots (although disengagement was defined as absence of positive and negative interaction). Positive behaviors were common throughout the observation period. There were very few (2.74%) snapshots that consisted of *only* negative behaviors. Such an infrequent code would have led to convergence problems in the analysis. Consequently we defined a snapshot as Negative when any display of negativity occurred. Disengagement was coded in the absence of negativity and positivity. The Positive, Negative and Disengaged codes were therefore mutually exclusive.

Coders were trained and were determined reliable with one another before independent coding began and throughout the coding period to prevent rater drift. The Kappas for Sibling 1 and Sibling 2 respectively were .71 and .81 for the Positive codes and .81 and .78 for the Negative codes. No Kappa is presented for Disengagement as it was defined as the absence of the other codes.

#### Mind orientated parenting: Maternal sensitivity

Observations of mother interaction with each child were gathered in the home using three tasks: (i) free play with no toys (5 minutes); (ii) structured play with mother teaching child; and (iii) the mother and child reading a wordless picture book together. Maternal sensitivity was assessed using the sensitive responding and mutuality scales of the Coding of Attachment-Related Parenting [42] as well as the positive control scale of the Parent-Child Interaction System (PARCHISY, [43]), rated on a 7 point scale. Internal consistency was *α* = 0.85; Inter-rater reliability, assessed by double coding 10% of tapes (throughout coding period) and assessed by Cronbach’s *α* [44] was 0.94.

#### Mind-oriented parenting: Reflective Capacity

This was made up of two components derived from the maternal interview:

- Children’s mental attributes. Mothers were asked to describe each of their children using the following prompt from the five minute speech sample [45], adapted in our study to three minutes: “Now, I’d like to hear your thoughts and feelings about ____ (insert child’s name) in your own words…I’d like you to speak for approximately three minutes, telling me what kind of person ___ (insert child’s name) is and how the two of you get along together.” Answers were audiotaped and transcribed. We counted the number of times mothers spoke about children’s cognitive states, desires and emotions (as coded in [46]) during the three-minute speech sample. As the number of mental attributes was higher with child age, r (666) = .12, p< .001, we residualized mental attributes for child age and then proportionalized these scores by word count to control for maternal loquacity.
- Reflective parenting. Mothers were asked “How have your experiences in your childhood affected you as a parent”? The scoring was based on the mothers’ ability to talk about both her early experience and her current parenting and her attention to thoughts and feelings within her answer. Our coding was guided by the work of Fonagy and Target [26]. A five point scale was used from no reflective parenting (0) to high reflective parenting (5).

Two coders were trained to criterion on these two components and then reliability was checked throughout the coding period on 10% of narratives. Inter-rater reliability for children’s mental attributes was *α* = 0.97, and reflective parenting was *α* = 0.80. These were correlated r (667) = .23, p<.001 and a composite was constructed.

#### Positivity

Mothers’ perceptions of the positive behaviors she directs towards each child were assessed using a scale from the National Longitudinal Survey of Children and Youth [47], adapted from Strayhorn and Weidman’s [48] Parent Practices Scale. Mothers rated five survey items (e.g., “How often do you do something together that he/she enjoys?”) for positivity on a five-point scale ranging from never (1) to almost always (5) and the mean across items was taken. The internal consistency of the scale is *α* = .79.

#### Demographics/Child Characteristics

Child age and gender was collected via parent reports. Specifically, mothers provided their children’s dates of birth. Age of older children was coded into two categories 0 = < 4.5 years, 1 = ≥ 4.5 years old). Gender of the target child was coded (0 = boy and 1 = girl) as was the gender composition of the sibling pair (boy-boy, boy-girl, girl-boy, girl-girl). To capture family Socioeconomic Status (SES), household income was reported by mothers and coded on a 16 point scale ranging from no income (1) to $105,000 or more (16) and was then standardized. Information on assets was collected in three areas: owning a home, a car and number of rooms in the household. Assets were standardized and composite scores were calculated. The correlation between income and assets was r (336) = .64, *p* < .001. A composite of income and assets was constructed by taking the mean of the two variables.

### Analytic plan

#### Growth mixture model for temporal sequences in behavior

In order to determine whether there are distinct temporal sequences in children’s behavior during sibling interaction we used GMM (e.g. [11], [12], [13]). This method reduces the dimensionality of data, which is essential given that we have a large number of observations (up to 30) per child and therefore many possible observed patterns of behavior.

Separate models were fit for younger and older children. The outcome variable is the behavior of a child in a 20-second segment (coded as positive, negative or disengaged). The nominal outcomes were analysed using a multinomial logit model consisting of two equations that contrast the probabilities of disengagement and negativity in each segment with the probability of positivity (the baseline category). In a multinomial GMM, the log-odds of disengagement versus positivity and of negativity versus positivity depend on time (time since the start of the interaction) and between child variation (variation between children that is not explained by factors in the model). Children are assumed to come from *K* latent subpopulations (or classes). Each class has a distinct temporal behavioral sequence in terms of its intercept, pattern of change over time, and the extent of between-child variation due to unmeasured factors. The goal of the analysis is to determine the number of latent classes and the nature of the temporal behavioral sequence (in terms of disengagement, negativity and positivity) within each class. Temporal behavioral sequences were found to be well represented by quadratic functions of time in each class and for both younger and older children. The models were estimated using the Mplus software [49]. Further details of the model specification are given in S1 File.

Deciding on the number of classes that represent the data is a difficult topic in growth mixture modeling. Two reviews [50][51] suggest that the sample-size adjusted BIC [52] and LMR statistic [53] tend to perform relatively strongly in extracting the correct number of classes. While such statistical criteria are useful, they can disagree on the correct number of classes. Thus, it is important to also decide the number of classes on the grounds of theory, parsimony and substantive interpretability. Where our statistical criteria disagree, we aid our identification of the preferred model by presenting and interpreting the results of the competing models.

Having established the preferred model as a K-class model, we further assess the fit of the model by examining the precision with which children might be classified into distinct classes. We do this by first assigning children to the classes to which they have the highest probabilities (their most likely classes) and then, for each class, we calculate the mean of these assignment probabilities. The higher these means, the more precisely children can be classified into classes.

#### Analysis of predictors of temporal behavior sequences

After identifying the patterns of temporal behavioral sequences for younger and older children, we examine predictors of class membership, treating the latent classes as categories of a nominal latent variable in a second multinomial logit model.

#### Analysis of association between siblings’ temporal behavioral sequences

To investigate the extent of reciprocity or complementarity in siblings’ behavior, we examine the association between the temporal behavioral sequences of sibling pairs. The younger child’s expected class was then cross-tabulated with the class of their older sibling. To allow for uncertainty in class membership, a simulation procedure was used to obtain a p-value for testing the association between sibling classes (see S1 File).

#### Missing data

Of the 397 families that took part at Time 2, 336 were observed in sibling interaction. We compared families that completed all observational tasks with those who did not on family income and assets. Families that did not complete all tasks compared to those who did, had fewer assets (including fewer rooms) (F (1, 395) = 7.4, p < .007, M = -.28 versus .05 and lower income (F (1, 395) = 7.3, p < .007, M = 11.7 versus 12.9. Not all sibling pairs were observed for the full 10 minutes as children wandered away from the camera and could not be coaxed back. A total of 244 children (73%) were observed for at least 25 of the 30 possible 20-second segments, with only seven (2%) observed over fewer than 10 segments. All 336 children contribute information to the estimation of the GMM under a ‘missing at random’ (MAR) assumption [54]. Thus the probability of dropout at time *t* may depend on time and children’s observed (pre-dropout) behavior, but not on behavior after dropout. The maximum likelihood method used to fit the GMM is an efficient way to use all the available data and is an alternative to multiple imputation which also assumes MAR [55]. In the analysis of the predictors of class membership, there is missing covariate information for 18% of families. Again, all information is incorporated under MAR using the maximum likelihood approach implemented in Mplus.

## Results

Sibling interaction was largely positive with children exhibiting only positive behaviors in 66% of the segments in which they were observed. Children exhibited negative behaviors in 22% of segments. They did not engage with their sibling in 12% of segments. Maternal sensitivity and maternal reflective capacity were significantly correlated with one another (r = 0.30, p<.05) and to SES (r = 0.37, p<.05 and r = 0.30, p<.05 respectively), but none of these were associated with maternal positivity. Gender composition did not predict group membership in older or younger siblings and was dropped from subsequent models.

### Younger siblings

#### Number and description of classes

Table 1 shows the model fit statistics, for younger siblings for 1, 2 and 3 class models. Most fit statistics point to a 2-class model. Although the sample size adjusted BIC is lowest for the 3-class model, the BIC is lowest for a 2-class model. Furthermore, the LMR test suggests that we should reject the one-class model in favour of the 2-class model (*p* = 0.037), but that the 3-class model is not a significant improvement over the 2-class model (*p* = 0.174).

**Table 1:** Fit Indices for Younger and Older Siblings for 1–4 Class Models.^†^

	1 class	2 classes	3 classes		
Younger siblings					
# parameters	9	19	29		
Log Likelihood	-7353	-7272	-7229		
LMR LRT ^‡^	-	**0.037**	0.174		
Adjusted BIC	14758	14656	**14628**		
BIC	14787	**14717**	14720		
	1 class	2 classes	3 classes	4 classes	5 classes
Older siblings					
# parameters	9	19	29	39	49
Log Likelihood	-6554	-6495	-6446	-6419	-6405
LMR LRT ^‡^	-	0.267	0.249	**0.064**	0.704
Adjusted BIC	13161	13102	**13062**	13068	13099
BIC	13190	13163	**13155**	13192	13255

*Note*.

^†^ For each fit index, the model with the preferred number of classes is highlighted in bold.

^‡^ *p*-values compare the current *K*-class model to the model with *K*-1 classes (H_0_). LMR LRT = Lo, Mendell & Rubin likelihood ratio test. Adjusted BIC = Sample size adjusted Bayesian Information Criterion

**Fig 1:**
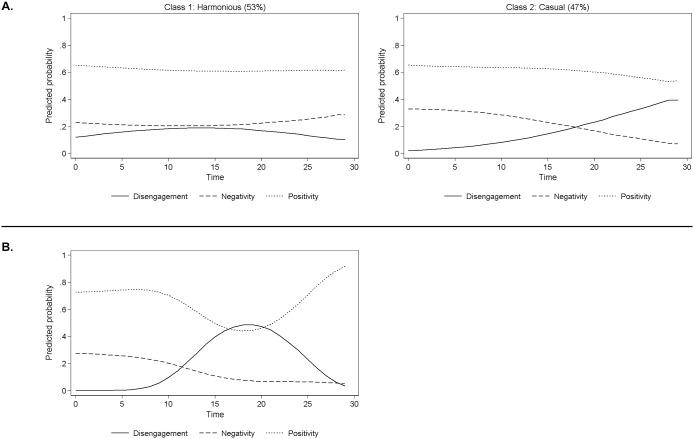
Average predicted probability of temporal behavioral sequences from the accepted 2-class model (A) and the third class from the rejected 3-class model (B) for younger children.

Readers not familiar with the statistical techniques may find it most helpful to refer to the plots given in Fig 1A and 1B. These show the typologies of temporal behavioral sequences that were identified, separately for the younger and older children. Time is on the x-axis. The lines can be understood as depictions of the likelihood of children showing positivity, negativity or disengagement within the 20-second snapshot. We named these typologies based on the pattern seen for positivity, negativity and disengagement. The temporal behavioral sequences plots for the 2-class model for younger siblings (Fig 1A) show that children in class 1 (53% of children) have a high probability of positivity and low probabilities of negativity and disengagement with little change over time. (See Table A1 in S1 File for the estimated coefficients of the quadratic functions that generated these plots). We call this class the *harmonious* group. Children in class 2 (47%) have a slightly lower but still stable probability of positivity. However, as interaction proceeds for children in class 2, the probability of negativity declines while the probability of disengagement increases substantially. We refer to this expected class as the *casual* group.

Although the BIC and LMR LRT statistics suggested that a 2-class model was an adequate fit to the data, as a robustness check, because the sample size adjusted BIC was lowest for the 3-class model, we also examined the fitted temporal behavioral sequences from a 3-class model. We found that the two classes that we saw in the 2-class model are still evident in the 3-class solution. The new third class (Fig 1B) includes children who show a period of deteriorating interaction (dip in positivity and increase in disengagement) with a subsequent recovery (increase in positivity and decrease in disengagement). Thirteen percent of children were in this *recovery* group. Although we settled on the 2-class model for further analysis, it is noteworthy that the third class does represent the *recovery* process discussed in the introduction and only hypothesized for older children. The mean posterior class membership probabilities can be seen for the 2-class model in the top of Table 2. These show that if a child is allocated to the class for which their class membership probability is highest, they have a greater than 80% chance of belonging to that class indicating that the two classes are well differentiated.

**Table 2:** Mean Posterior Class Membership Probabilities for Younger and Older Siblings by Most Likely Class.

Younger siblings		Mean posterior class membership probability			
		Harmonious	Casual		
Most likely class	1	0.888	0.112		
	2	0.179	0.821		
Older siblings		Mean posterior class membership probability			
		Recovery	Harmonious	Casual	Deteriorating
Most likely class (4-class model)	1	0.748	0.079	0.079	0.095
	2	0.075	0.725	0.075	0.124
	3	0.079	0.061	0.758	0.102
	4	0.065	0.104	0.078	0.752

#### Predictors of class membership

As hypothesized, mothers in the *casual* group showed lower levels of sensitivity and reflective capacity than mothers in the harmonious group (see Table 3). Contrary to expectation, SES was higher and more older siblings were older than 4.5 years of age in the *casual* group compared to the *harmonious* group.

**Table 3:** Covariate Effects on Class Membership for Younger and Older Siblings.

	Younger Siblings			Older Siblings								
	Harmonious			Recovery			Harmonious			Casual		
Parameter	Est.	SE	*p*-value	Est.	SE	*p*-value	Est.	SE	*p*-value	Est.	SE	*p*-value
Intercept	-1.005	0.349	0.004	-2.721	0.462	<0.001	-0.796	0.394	0.043	0.326	0.0408	0.425
Maternal sensitivity	0.172	0.039	<0.001	0.219	0.053	<0.001	0.148	0.059	0.003	-0.240	0.051	<0.001
Maternal reflective capacity	0.286	0.047	<0.001	-0.052	0.065	0.424	-0.024	0.061	0.682	-0.362	0.060	<0.001
Maternal positivity	0.115	0.074	0.119	0.310	0.089	<0.001	-0.012	0.080	0.880	0.044	0.081	0.589
SES	-0.136	0.042	0.001	0.315	0.065	<0.001	0.060	0.052	0.247	0.284	0.058	<0.001
Girl	0.063	0.057	0.266	-0.154	0.084	0.067	0.089	0.078	0.250	-0.074	0.081	0.357
Oldest child age 4.5 years or above	-0.199	0.062	0.001	0.308	0.093	0.001	0.091	0.085	0.284	0.427	0.087	<0.001

Note. *Disengaging* for younger children and *Deteriorating* for older children are the reference category in each analysis. Estimates reported on log-odds scale.

### Older siblings

#### Number and description of classes

**Fig 2:**
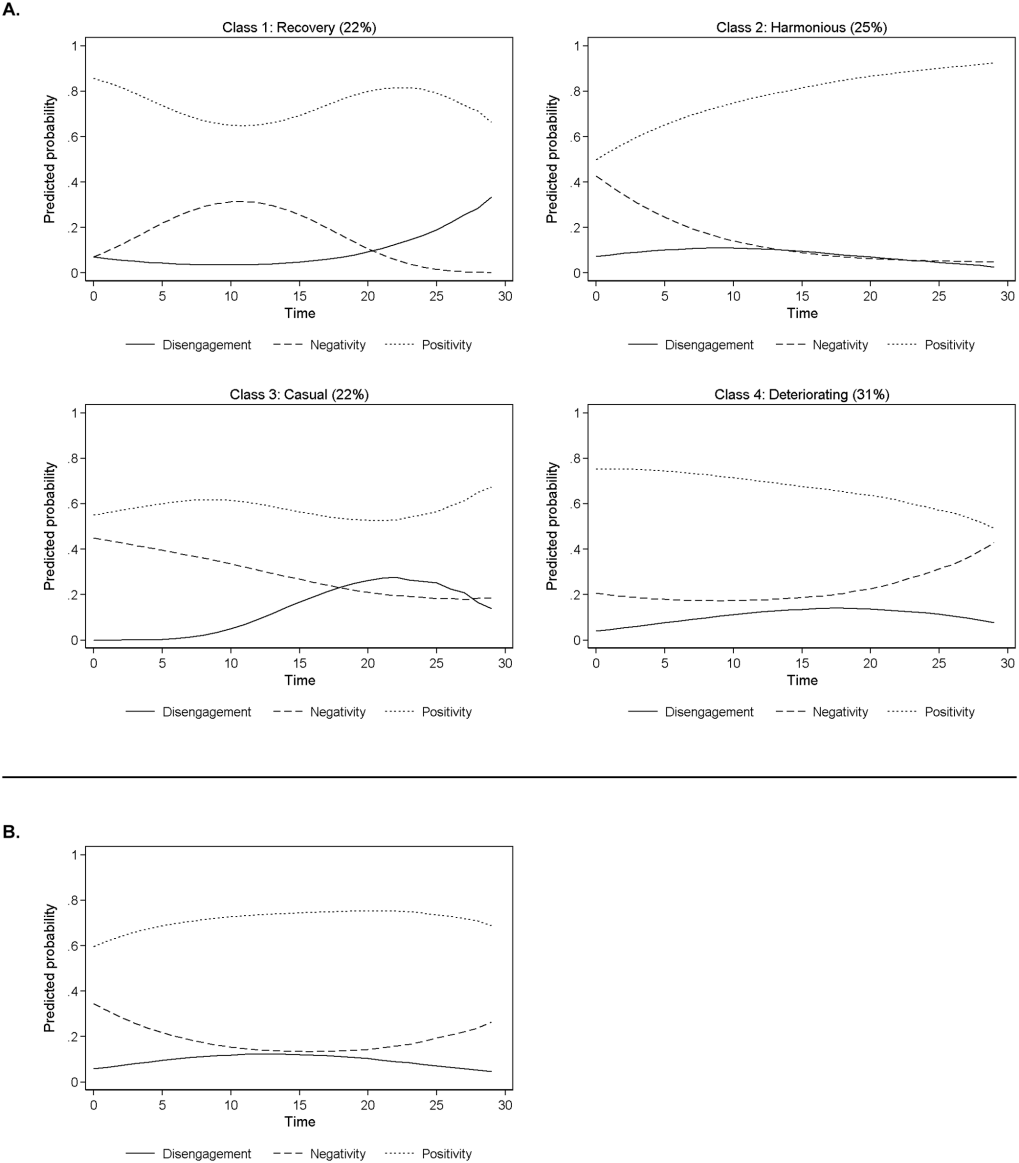
Average predicted probability of temporal behavioral sequences from the 4-class model for older children (A) and Class 2 of the rejected 3-class model for older siblings (B).

Model fit statistics for 1, 2, 3, 4 and 5 class models are provided in Table 1. The fit statistics suggest that we choose between a 3 and 4 class model. The BIC is lowest for the 3-class model. The LMR LRT, however, suggests we cannot reject two classes in favour of three classes (*p* = 0.249), but we come close to rejecting three classes in favour of four classes (*p* = 0.064). Thus the BIC suggests a 3-class model whereas the LMR LRT suggests that a fourth class may provide a significantly better fit. Although the statistical criteria suggest that there are discrete temporal behavioral sequences of child behavior, they do not agree conclusively on the number of discrete classes to best represent the data. In the 3-class model for older siblings we see a group that combines two processes hypothesized in the introduction: harmonious and deteriorating interaction (increased negativity). This combined class is presented in Fig 2. In the 4-class model this class split into two, revealing a harmonious group and a deteriorating interaction group. As the fit statistics were ambiguous between the 3- and 4-class models and because we had hypothesized that these two processes would represent separate classes we opted for the 4-class solution.

The four groups can be seen in Fig 2A (and the estimated coefficients for the quadratic functions in time are in Table A2 of S1 File). The first class includes 22% of children. In this group children start well (with a high probability of positivity and low probability of negativity), run into a problem (positivity drops, negativity increases) but recover (positivity and negativity drops). Their disengagement goes up only at the end of the interaction. We call this group the *recovery* group. The next group included 25% of children. Children in this group increased their probability of being positive and decreased their probability of being negative over time. We call this the *harmonious* class. The third group represented 22% of children. Children in this group begin with a relatively high probability of negativity (compared to positivity) which drops over the course of the interaction. Disengagement, however, begins early (one-third of the way into the interaction) and the children remain disengaged for the rest of the interaction. We call this the *early disengagement group*. The fourth group included 31% of children. In this group children show a high probability of positivity at the start of the interaction, but increase in negativity as the interaction proceeds. We call this the *deteriorating* group. In summary we found support for the hypothesis that for older siblings there would be deteriorating, harmonious and recovery groups. We also found support for the presence of a *casual* group among older siblings. The mean posterior class membership probabilities can be seen for the 4-class model in the bottom of Table 2. The four classes are well-differentiated from one another, although slightly less well differentiated than in younger children. For all groups we accurately allocate around 75% of children to classes (compared to the over 80% for younger children).

#### Predictors of class membership

As the reference group the *deteriorating* group was found to differ significantly from the other three groups (see Table 3). Mothers of children in the *harmonious* group were significantly more sensitive than mothers of children in the *deteriorating* group. Mothers of children in the *recovery* group were significantly more sensitive and positive and were higher in SES than mothers in the deteriorating group. Children were also older than those in the *deteriorating* interaction group. Mothers of children in the *casual* group showed lower sensitivity and reflective capacity, came from higher SES backgrounds and their older, children were more likely to be older than 4.5 years of age when compared to mothers with children in the *deteriorating* group.

### Similarity between siblings’ temporal behavioral sequences

Table 4 shows the cross-tabulation of the classes of behavioral sequences of both siblings, after assigning children to the class with the highest probability of membership. Using the simulation approach described earlier to take account of uncertainty in class membership, the association is statistically significant (p<0.001). If the older child is *harmonious* or is in the *casual* class, the younger child is more likely than expected to be in the *casual* class. If the older child *deteriorates*, the younger child is more likely than expected to be *harmonious*.

**Table 4:** Association between Modal Classes for Younger and Older Siblings.

	Older sibling				
	Recovery	Harmonious	Casual	Deteriorating	Total
Younger siblings					
Harmonious	34	36	22	73	165
	*48*.*6*	*40*.*4*	*29*.*7*	*70*.*9*	*49*.*1*
Deteriorating/ Disengaging	36	53	52	30	171
	*51*.*4*	*59*.*6*	*70*.*3*	*29*.*1*	*50*.*9*
Total	70	89	74	103	336
	*100*.*0*	*100*.*0*	*100*.*0*	*100*.*0*	*100*.*0*

*Note*. Numbers in cells are frequencies and *column percentages*

## Discussion

Children in this sample engaged in sustained, largely positive interactions. Average use of strategies during interactions has been reported elsewhere [56]. Yet, we know that social interaction is sequential and that simply looking at behavior in aggregate may mask meaningful individual differences in interaction styles [57]. This study is unique in that we characterized temporal behavioral sequences in young siblings’ interactions.

### Typologies of sibling interaction based on temporal sequences

This study provides evidence of discrete subtypes of temporal sequences of behavior for young children who are interacting with a sibling. These discrete behavioral sequences were found for both older and younger siblings in our sample based on positivity, negativity and disengagement. Using a combination of statistical criteria and substantive interpretation we concluded that the behavioral patterns of the younger children in our sample were best characterized by two classes while the older children’s patterns were best characterized by four classes. As we expected, one group in both the older and the younger cohorts exhibited a *harmonious* pattern. Many more (53%) of the younger children exhibited this pattern, compared to the older children (25%). It is worth noting that while the *harmonious* group of younger children remained highly stable across the observation period, the *harmonious* group of older children actually increased in positivity and decreased in negativity over time.

We also found support for the presence of a *recovery* group amongst the older siblings. The quality of these children’s interaction declined about three minutes into the observation but they quickly recovered and returned to their high probability of positivity versus negativity. Given that young siblings do oppose and provoke one another [58,59] the ability to recover from minor altercations is likely to be very important in enabling siblings to interact positively with one another. The 3-class model for young siblings revealed a similar recovery category exhibited by few (13%) younger siblings. The model fit statistics, parsimony principle and our hypotheses did not support interpretation of this third class in the younger children but a longitudinal analysis of the development of this pattern is worth pursuing.

Nearly one-third (31%) of the older children displayed the expected deteriorating behavioral sequence. Both older and younger siblings displayed a *casual* group, although as expected, these manifested themselves somewhat differently. Half of the younger children showed a pattern whereby they disengaged while remaining fairly positive and even showing declines in negativity. Older children in the *casual* category showed a spike in disengagement earlier in the interaction and were less positive and more negative throughout. These older children may simply not be interested in interacting with their younger sibling, but they are not negative towards them. As discussed earlier, the relative lower power status of younger siblings may make them reluctant to direct/reciprocate negativity towards their more powerful older sibling [33], [32]. For adults, disengaging (e.g., ignoring or withdrawing) during an interaction is considered escalatory [60]. For young children such strategies may reflect an adaptive way of extracting themselves from an interaction that is becoming stressful. For example, Perlman and Ross [61] found that rates of ignoring (along with other oriented reasoning and compliance) during conflicts between preschool aged siblings were higher after parents intervened suggesting that disengagement may actually be relatively adaptive. In our study, compared to the *harmonious* group, younger children in the *casual* group had mothers who were less sensitive and had poorer reflective capacity. Older children in the *early disengagement* group, compared to children from the *deteriorating* group, came from homes with less sensitive and less reflective mothers (although they had higher SES). Thus, our findings suggest that for young siblings who are frequent playmates disengaging may reflect poorer functioning than a deteriorating pattern. A closer examination of the psychological meaning of disengagement across development is needed to better understand its impact.

The younger siblings in our study are substantially younger than the participants on which previous taxonomies were based. The fact that their behaivour is less differentiated (i.e. it was best characterized by two classes) suggests that differentiation may develop as children mature. For the older children, *recovery* and *casual* may well have been identified as one category if the taxonomy was based on averages. Attending to the temporal sequence, however, resulted in a more refined characterization of older siblings that suggested that some older siblings do know how to recreate a positive tone in the interaction, when it has been lost.

### What child and family characteristics were the different classes related to?

#### Maternal mind-oriented parenting and positivity

We expected that children whose mothers were more mind-oriented and positive would be more harmonious and more effective at repairing the quality of their interaction if it began to disengage/deteriorate. Overall, we found support for our hypothesis in that maternal behavior was related to the behavioral sequences children exhibited when interacting with their sibling. Maternal positivity has received a great deal of attention in the developmental literature [62], [63]. Maternal mind-oriented parenting (i.e., the capacity to get into the mind of the child) is less well explored [64], [24]. This is the first study to suggest that a family environment in which mothers think about and are responsive to the mental states of their children, may foster a benign interactional sequence between siblings that is either harmonious or includes the capacity for repair if the interaction deteriorates. Of course, causal links between parent and child behavior are unwarranted in this study given that it was not genetically sensitive or longitudinal. It is possible that sibling interactions influence maternal mind-oriented parenting and positivity. It is also possible that shared genes between parents and children influence mind-oriented parenting, positivity and their association with sibling behavior.

#### Age and age gap between siblings

Based on differences in emotional regulation [31] we expected, and found, that some older children exhibit a *recovery* behavioral sequence. Counter to our expectation, for the younger children in our sample, having an older sibling who was closer to their own age (i.e., younger) was associated with use of more adaptive behavioral sequences (i.e., *harmonious* vs. *casual*). Based on a sample of middle childhood and adolescent children, Buhrmester and Furman [65] reported greater intimacy for siblings who are closer in age. Perhaps for children in our sample being close in age increased the motivation to remain engaged. However, for the older siblings, a larger age gap was associated with the *recovery* and *casual* classes. This finding may be explained by the fact that in our sample, the age of the older sibling is confounded with the age gap between the children. Findings about the impact of age gap have been weak and inconsistent [66] and more research is needed to explore whether and how they influence family dynamics.

#### Socioeconomic status (SES)

Children in the *recovery* and *casual* groups came from higher SES homes when compared to children in the *deteriorating* group. For the older children, coming from homes with more resources is associated with the use of more adaptive temporal behavioral sequences during sibling interaction. Younger children in the *harmonious* group came from lower SES homes than children in the *casual* group. Few studies have explored the link between SES and the quality of sibling interaction. Those that have, defined SES differently than we did (e.g., Kretschmer and Pike [23] used a measure of maternal education) and examined overall interaction quality, not behavioral sequences. Thus, more research is needed to understand the potential link between family demographics and sibling interaction quality.

### Similarity between children’s temporal behavioral sequences during sibling interactions

The limited past research in this area suggests that children tend to reciprocate one another’s actions [36], [4]. Our finding of 4 classes for older children and 2 classes for younger children and the common pairings we observed argue against reciprocity. For example, 71% of older siblings with a deteriorating pattern have a sibling who was classified as harmonious. Adopting a harmonious temporal behavioral sequence may be highly adaptive when interacting with an older sibling who is becoming more negative. We did see some support for reciprocity specifically around disengagement. Of those older children who were in the *casual* class, 70% of their siblings also identified as being in that group, suggesting the tit-for-tat pattern that Perlman and Ross [4] describe. However, our analyses do not allow us to disentangle the direction of these effects. Despite this limitation and counter to our expectations, our findings suggest more complementarity, not reciprocity, in the pairings of sibling temporal behavioral sequences.

GMM represents a promising way to deal with the dimensionality of moment-to-moment interaction data. However, our results illustrated the ambiguity that can arise when using multiple fit statistics and theoretical concerns to determine the optimal number of classes. For example, although most fit statistics suggested a 2-class solution for the younger children, the 3-class solution yielded a group (the *recovery* group) that, although not expected, made sense. In keeping with recommendations by Muthen (2003) [67] we engaged in “substantive checking” as a way of dealing with disagreement among statistical indicators of the appropriate number of classes. This involves using predicators to test the validity of specific classes and aid in the interpretation of the appropriate number of classes. This issue of ambiguity is not limited to this study and has been discussed extensively elsewhere [50], [51].

A second issue relates to the fact that all of our dyads interacted for no more than ten minutes. Although short observation periods for family interaction are widely used (e.g., [68]), it is possible that our observation period captures only the early phase of real-world sibling interactions. This seems unlikely given that the ‘validity’ of the classes was established through their association with hypothesized predictors. Balancing resource demands by relying on limited observation periods vs. having larger samples sizes is a challenge. It will be important, although very labor intensive, in future studies to compare class extraction based on different periods of observation.

In this study we fitted a separate growth mixture model (GMM) for each sibling to investigate their behavior trajectories over the course of an interaction, which has the advantage of allowing different latent classes to be defined for each child. At some point in the future it may be possible to fit a parallel process GMM in which siblings’ behaviors are modeled jointly and a common set of latent classes is defined by the temporal sequences of both siblings. These models are too complex to be fit without convergence problems at the moment using existing software. It is not possible to combine the three components of 1) individuals interacting in a dyad, 2) temporality and 3) causal influence (e.g. tit for tat) in one model. Furthermore, nominal versus continuous variables add further complexity to this endeavor. Although there are examples of models that include one component there are none that combine two or more components [69]. Further potential extensions are discussed in S1 File.

Based on repeated interactions with their environments children are thought to develop cognitive structures that come to guide their interactions with others. Internal working models of attachment are one key mechanism [70] but it does not address the sequential aspect of interaction. Script theory [71] posits that based on repeated experiences individuals develop routine, and sequential, ways of behaving. This idea has received little attention from researchers studying interactions. Based on such thinking we speculate that children should develop routine behaivoural patterns of interaction that come to guide subsequent behavour. This would suggest that these patterns should become stable over time and may spill over across interaction partners. We hope that by using a methodology such as the one described in this paper that it will eventually be possible to test whether children exhibit complex sequential patterns across time and interaction partners.

We speculate that the different classes identified in our study will be related to relationship outcomes in ways that differ from those predicted by the typologies of sibling relationships identified using aggregate data. One point of differentiation may be in children’s perceptions of, and satisfaction with, their sibling relationship. For example, the older children in the *recovery* class are likely to be more satisfied with their relationship than children in the *casual* or the *deteriorating* groups, even though average positivity/negativity in these groups may not be very different in aggregate. We hope to collect such data from the children in our sample when they are old enough to provide it. We also speculate that these patterns may spill over into other relationships in the way that internal working models come to govern subsequent relationships.

This study provides an important first step in using an existing methodology to answer a novel question. Exploring the different types of sequences exhibited by a diverse sample of children living in a large urban center provides a deeper understanding of children’s interactions. The benefits of our approach become evident when considering the distinctiveness of the four patterns older children exhibited. Average rates of positivity/negativity and disengagement, or even a temporal behavioral sequences based on the entire sample would have masked important differences between children, differences that are likely to have implications for children’s subjective experience of sibling interactions. Our findings also highlight the relationship between mother’s mind-oriented parenting, maternal positivity and children’s behavioral sequences: children who experience more positive parenting show more adaptive temporal sequences. Finally, our findings suggest that complementary rather than reciprocity is a key pattern in the interactions of young siblings.

## Supporting Information

S1 File Additional details on statistical methods. (DOCX)

S2 File Sequences of sib interaction data. (CSV)

## References

[1] Pike A, Coldwell J, Dunn JF. Sibling relationships in early/middle childhood: links with individual adjustment. J Fam Psychol. 2005;19: 523–532. 10.1037/0893-3200.19.4.523 16402867

[2] Dunn J, Munn P. Development of justification in disputes with mother and sibling. Dev Psychol. 1987;23: 791–798. 10.1037/0012-1649.23.6.791

[3] Patterson G. Coercive family processes. Eugene, OR: Castalia Pub. Co; 1982.

[4] Perlman M, Ross HS. If-Then Contingencies in Children’s Sibling Conflicts. Merrill-Palmer Q. 2005;51: 42–66. 10.1353/mpq.2005.0007

[5] Stoolmiller M, Snyder J. Modeling heterogeneity in social interaction processes using multilevel survival analysis. Psychol Methods. 2006;11: 164–177. 10.1037/1082-989X.11.2.164 16784336

[6] Kramer L, Perozynski LA, Chung T-Y. Parental Responses to Sibling Conflict: The Effects of Development and Parent Gender. Child Dev. 1999;70: 1401–1414. 10.1111/1467-8624.00102 10621963

[7] Ross H, Ross M, Stein N, Trabasso T. How Siblings Resolve Their Conflicts: The Importance of First Offers, Planning, and Limited Opposition. Child Dev. 2006;77: 1730–1745. 10.1111/j.1467-8624.2006.00970.x 17107457

[8] Messman S, Canary D. Patterns of conflict in personal relationships In: Spitzberg BH, Cupach WR, editors. The dark side of close relationships. Mahwah, N.J: Lawrence Erlbaum Associates; 1998 pp. 121–152.

[9] Singer JD, Willett JB. Applied longitudinal data analysis: modeling change and event occurrence. Oxford; New York: Oxford University Press; 2003.

[10] Nagin DS. Analyzing developmental trajectories: A semiparametric, group-based approach. Psychol Methods. 1999;4: 139–157. 10.1037/1082-989X.4.2.139 11285809

[11] Jung T, Wickrama KAS. An Introduction to Latent Class Growth Analysis and Growth Mixture Modeling. Soc Personal Psychol Compass. 2008;2: 302–317. 10.1111/j.1751-9004.2007.00054.x

[12] Muthén B, Asparouhov T. Growth mixture modeling: Analysis with non-Gaussian random effects In: Fitzmaurice GM, Davidian M, Verbeke G, Molenberghs G, editors. Longitudinal data analysis. Boca Raton: CRC Press; 2009 pp. 143–165.

[13] Vermunt J. Growth models for categorical response variables: Standard, latent-class, and hybrid approaches In: van Montfort K, Oud J, Satorra A, editors. Longitudinal models in the behavioral and related sciences. New Jersey: Lawrence Erlbaum; pp. 139–158.

[14] McGuire S, McHale SM, Updegraff K. Children’s perceptions of the sibling relationship in middle childhood: Connections within and between family relationships. Pers Relatsh. 1996;3: 229–239.

[15] McHale SM, Whiteman SD, Kim J-Y, Crouter AC. Characteristics and correlates of sibling relationships in two-parent African American families. J Fam Psychol. 2007;21: 227 10.1037/0893-3200.21.2.227 17605545

[16] Gamble WC, Yu JJ. Young Children’s Sibling Relationship Interactional Types: Associations with Family Characteristics, Parenting, and Child Characteristics. Early Educ Dev. 2014;25: 223–239. 10.1080/10409289.2013.788434

[17] Stewart RB, Verbrugge KM, Beilfuss MC. Sibling relationships in early adulthood: A typology. Pers Relatsh. 1998;5: 59–74.

[18] Stewart RB, Kozak AL, Tingley LM, Goddard JM, Blake EM, Cassel WA. Adult sibling relationships: Validation of a typology. Pers Relatsh. 2001;8: 299–324.

[19] Whiteman SD, Loken E. Comparing analytic techniques to classify dyadic relationships: An example using siblings. J Marriage Fam. 2006;68: 1370–1382.

[20] Brody GH, Stoneman Z, McCoy JK. Contributions of family relationships and child temperaments to longitudinal variations in sibling relationship quality and sibling relationship styles. J Fam Psychol. 1994;8: 274–286. 10.1037/0893-3200.8.3.274

[21] Dunn J Sibling relationships In: Smith PK, Hart CH, editors. Blackwell handbook of childhood social development. Oxford, UK; Malden, MA: Blackwell Publishers; 2002.

[22] Jenkins J, Rasbash J, Leckie G, Gass K, Dunn J. The role of maternal factors in sibling relationship quality: a multilevel study of multiple dyads per family: Multilevel study of sibling relationship quality. J Child Psychol Psychiatry. 2012;53: 622–629. 10.1111/j.1469-7610.2011.02484.x 22141370

[23] Kretschmer T, Pike A. Young children’s sibling relationship quality: distal and proximal correlates. J Child Psychol Psychiatry. 2009;50: 581–589. 10.1111/j.1469-7610.2008.02016.x 19236529

[24] Meins E, Fernyhough C, Wainwright R, Das Gupta M, Fradley E, Tuckey M. Maternal Mind-Mindedness and Attachment Security as Predictors of Theory of Mind Understanding. Child Dev. 2002;73: 1715–1726. 10.1111/1467-8624.00501 12487489

[25] Smith J, Ross H. Training Parents to Mediate Sibling Disputes Affects Children’s Negotiation and Conflict Understanding. Child Dev. 2007;78: 790–805. 10.1111/j.1467-8624.2007.01033.x 17517005

[26] Fonagy P, Target M. Attachment and reflective function: Their role in self-organization. Dev Psychopathol. 1997;9 10.1017/S0954579497001399 9449001

[27] Cutting AL, Dunn J. Conversations with siblings and with friends: Links between relationship quality and social understanding. Br J Dev Psychol. 2006;24: 73–87. 10.1348/026151005X70337

[28] Bowlby J. Attachment and loss. 2nd ed New York: Basic Books; 1999.

[29] Kochanska G, Aksan N, Carlson JJ. Temperament, relationships, and young children’s receptive cooperation with their parents. Dev Psychol. 2005;41: 648–660. 10.1037/0012-1649.41.4.648 16060811

[30] Eisenberg N, Cumberland A, Spinrad TL, Fabes RA, Shepard SA, Reiser M, et al The relations of regulation and emotionality to children’s externalizing and internalizing problem behavior. Child Dev. 2001;72: 1112–1134. 11480937 10.1111/1467-8624.00337

[31] Eisenberg N, Hofer C, Vaughan J. Effortful control and its socioemotional consequences In: Gross J, editor. Handbook of emotion regulation. New York: Guildford Press; 2007.

[32] Schvaneveldt J, Ihinger M. Sibling relationships in the family In: Nye F, Reiss I, editors. Contemporary theories about the family. New York: The Free Press; 1979 pp. 453–467.

[33] Perlman M, Siddiqui A, Ram A, Ross H. An Analysis of Sources of Power in Children’s Conflict Interactions In: Mills R, Duck S, editors. Developmental Psychology of Personal Relationships. Chichester: Wiley; 1999.

[34] Fischer A, Manstead A. Functions of emotion from an organizational perspective In: Ashkanasy NM, Cooper CL, editors. Research companion to emotion in organizations. Cheltenham, UK; Northampton, MA: Edward Elgar; 2008.

[35] Howe N, Ross HS, Recchia H. Sibling Relations in Early and Middle Childhood In: Smith PK, Hart CH, editors. The Wiley-Blackwell Handbook of Childhood Social Development. Oxford, UK: Wiley-Blackwell; 2010 pp. 356–372. Available: http://doi.wiley.com/10.1002/9781444390933.ch19

[36] Phinney JS. The Structure of 5-year-olds’ Verbal Quarrels with Peers and Siblings. J Genet Psychol. 1986;147: 47–60. 10.1080/00221325.1986.9914479

[37] Vuchinich S. Sequencing and Social Structure in Family Conflict. Soc Psychol Q. 1984;47: 217 10.2307/3033819

[38] Rasbash J, Jenkins J, O’Connor TG, Tackett J, Reiss D. A social relations model of observed family negativity and positivity using a genetically informative sample. J Pers Soc Psychol. 2011;100: 474–491. 10.1037/a0020931 21341926

[39] Graaf R de, Bijl RV, Smit F, Ravelli A, Vollebergh WAM. Psychiatric and Sociodemographic Predictors of Attrition in a Longitudinal Study The Netherlands Mental Health Survey and Incidence Study (NEMESIS). Am J Epidemiol. 2000;152: 1039–1047. 10.1093/aje/152.11.1039 11117613

[40] Volling BL, McElwain NL, Miller AL. Emotion regulation in context: the jealousy complex between young siblings and its relations with child and family characteristics. Child Dev. 2002;73: 581–600. 11949910 10.1111/1467-8624.00425

[41] Howes C, Wu F. Peer Interactions and Friendships in an Ethnically Diverse School Setting. Child Dev. 1990;61: 537–541. 10.1111/j.1467-8624.1990.tb02798.x 2344788

[42] Matias C, Scott S, O’Connor T. Coding of Attachment-Related Parenting (CARP). London, UK: Institute of Psychiatry, Kings College; 2006.

[43] Deater-Deckard K, Pylas M, Petrill S. Parent-Child Interaction System (PARCHISY). London, UK: Institute of Psychiatry; 1997.

[44] Stemler S, Tsai J. Best practices in estimating interrater reliability In: Osborne J, editor. Best practices in quantitative methods. CA: Sage publications; 2008.

[45] Magaña AB, Goldstein MJ, Karno M, Miklowitz DJ, Jenkins J, Falloon IRH. A brief method for assessing expressed emotion in relatives of psychiatric patients. Psychiatry Res. 1986;17: 203–212. 10.1016/0165-1781(86)90049-1 3704028

[46] Jenkins JM, Turrell SL, Kogushi Y, Lollis S, Ross HS. A longitudinal investigation of the dynamics of mental state talk in families. Child Dev. 2003;74: 905–920. 12795397 10.1111/1467-8624.00575

[47] National Longitudinal Survey of Children and Youth (NLSCY). User’s handbook. Ottawa, Canada: Statistics Canada and Human Resources Development Canada; 1995.

[48] Strayhorn JM, Weidman CS. A Parent Practices Scale and Its Relation to Parent and Child Mental Health. J Am Acad Child Adolesc Psychiatry. 1988;27: 613–618. 10.1097/00004583-198809000-00016 3182627

[49] Muthén L, Muthén B. Mplus User’s Guide. Sixth Edition Los Angeles, CA; 1998 10.1037/met0000028

[50] Nylund KL, Asparouhov T, Muthén BO. Deciding on the Number of Classes in Latent Class Analysis and Growth Mixture Modeling: A Monte Carlo Simulation Study. Struct Equ Model Multidiscip J. 2007;14: 535–569. 10.1080/10705510701575396

[51] Tofighi D, Enders C. Identifying the correct number of classes in growth mixture models In: Hancock G, Samuelsen K, editors. Advances in latent variable mixture models. Greewich, CT: Information Age; 2008 pp. 317–341.

[52] Sclove SL. Application of model-selection criteria to some problems in multivariate analysis. Psychometrika. 1987;52: 333–343. 10.1007/BF02294360

[53] Lo Y, Mendell N, Rubin D. Testing the number of components in a normal mixture. Biometrika. 2001;88: 767–778. 10.1093/biomet/88.3.767

[54] Little R, Rubin D. Statistical Analysis with Missing Data. 2nd ed. New York: Wiley;

[55] Schafer JL, Graham JW. Missing data: Our view of the state of the art. Psychol Methods. 2002;7: 147–177. 10.1037/1082-989X.7.2.147 12090408

[56] Dunn J, Munn P. Siblings and the Development of Prosocial Behaviour. Int J Behav Dev. 1986;9: 265–284. 10.1177/016502548600900301

[57] Perlman M, Ross HS, Garfinkel DA. Consistent patterns of interaction in young children’s conflicts with their siblings. Int J Behav Dev. 2009;33: 504–515. 10.1177/0165025409343745

[58] Perlman M, Ross HS. The Benefits of Parent Intervention in Children’s Disputes: An Examnation of Concurrent Changes in Children’s Fighting Styles. Child Dev. 1997;68: 690–700. 10.1111/j.1467-8624.1997.tb04230.x

[59] Dunn J, Munn P. Siblings and the Development of Prosocial Behaviour. Int J Behav Dev. 1986;9: 265–284. 10.1177/016502548600900301

[60] Gottman JM. The roles of conflict engagement, escalation, and avoidance in marital interaction: A longitudinal view of five types of couples. J Consult Clin Psychol. 1993;61: 6–15. 10.1037/0022-006X.61.1.6 8450108

[61] Perlman M, Ross HS. The Benefits of Parent Intervention in Children’s Disputes: An Examnation of Concurrent Changes in Children’s Fighting Styles. Child Dev. 1997;68: 690–700. 10.1111/j.1467-8624.1997.tb04230.x

[62] Pettit GS, Bates JE, Dodge KA. Supportive parenting, Ecological Context, and Children’s Adjustment: A seven-Year Longitudianl Study. Child Dev. 1997;68: 908–923. 10.1111/j.1467-8624.1997.tb01970.x 29106716

[63] Davidov M, Grusec JE. Multiple pathways to compliance: Mothers’ willingness to cooperate and knowledge of their children’s reactions to discipline. J Fam Psychol. 2006;20: 705–708. 10.1037/0893-3200.20.4.705 17176207

[64] Fonagy P, Target M. Mentalization and the changing aims of child psychoanalysis. Psychoanal Dialogues. 1998;8: 87–114. 10.1080/10481889809539235 9572715

[65] Buhrmester D, Furman W. Perceptions of Sibling Relationships during Middle Childhood and Adolescence. Child Dev. 1990;61: 1387–1398. 10.1111/j.1467-8624.1990.tb02869.x 2245732

[66] Dunn J. Sibling relationships in early childhood. Child Dev. 1983;54: 787–811.

[67] Muthén B. Statistical and Substantive Checking in Growth Mixture Modeling: Comment on Bauer and Curran (2003). Psychol Methods. 2003;8: 369–377. 10.1037/1082-989X.8.3.369 14596497

[68] Kramer L, Gottman JM. Becoming a sibling: “With a little help from my friends”. Dev Psychol. 1992;28: 685–699. 10.1037/0012-1649.28.4.685

[69] Wu J, Witkiewitz K, McMahon RJ, Dodge KA, Conduct Problems Prevention Research Group. A parallel process growth mixture model of conduct problems and substance use with risky sexual behavior. Drug Alcohol Depend. 2010;111: 207–214. 10.1016/j.drugalcdep.2010.04.013 20558013 PMC2950227

[70] Bowlby J. Attachment and loss. 2nd ed. New York: Basic Books; 1999.

[71] Abelson RP. Psychological status of the script concept. Am Psychol. 1981;36: 715–729.

