# Supplementary material for: Capturing the Temporal Sequence of Interaction in Young Siblings
Source: PLoS One. 2015 May 21;10(5):e0126353. doi: 10.1371/journal.pone.0126353 (PMC4440720; doi:10.1371/journal.pone.0126353)
Supplement: S1 File — (DOCX) [file pone.0126353.s001.docx]

**Supplemental Information: Additional details on statistical methods**

**1. Additional details for the analysis of the association between siblings’ trajectories**

We tested for the association between the trajectories of sibling pairs using a simulation procedure to allow for uncertainty in class membership. This involved generating 1000 cross-tabulations from the posterior probabilities of class membership for each child, and calculating a chi-square test statistic and p-value for each table (in the usual way). The simulation-based p-value was calculated as the median p-value over the 1000 tables.

**2. Additional details and results for the growth mixture model**

The behavior of child *i* in a 20-second segment *t* is categorized into three mutually exclusive states, denoted by $y_{ti}$ where

$$y_{ti}=\left\{ \begin{matrix} 1 & \text{Disengagement} \\ 2 & \text{Negativity} \\ 3 & \text{Positivity} \end{matrix} \right.$$

and the multinomial response model consists of two equations contrasting the log-odds of disengagement versus positivity and negativity versus positivity. Children are assumed to come from $K$ latent subpopulations (or classes) with the class of child $i$ denoted by $C_{i}$. Each class has a distinct behavioral trajectory in terms of its intercept, time dependency and the extent of between-child variation. Various specifications of the dependency of behavior on time (including polynomials and step functions) were considered, including allowing different functions in different classes. Trajectories were found to be well represented by quadratic functions in each class and for both younger and older children. We therefore fitted multinomial growth mixture models of the following form:

$\log\left\{ \frac{\Pr\left( y_{ti}=r|C_{i}=k \right)}{\Pr\left( y_{ti}=3|C_{i}=k \right)} \right\}=\beta_{0k}^{\left( r \right)}+\beta_{1k}^{\left( r \right)}t+\beta_{2k}^{\left( r \right)}t^{2}+u_{ik}^{\left( r \right)}, \left( r=1,2;k=1,\ldots,K \right)$ $(1)$

where $\beta_{0k}^{\left( r \right)}$ is the intercept for class *k* in the equation for behavior *r* (disengagement or negativity) versus the reference behavior positivity, which indicates the initial log-odds of behavior *r* for children in class *k* (since *t* = 0 corresponds to the start of observation); $\beta_{1k}^{\left( r \right)}$and $\beta_{2k}^{\left( r \right)}$ together define the subsequent rate of change in the log-odds of behavior *r* for children in class *k*; and $u_{ik}^{\left( r \right)}$ is a normally distributed child-specific random effect which represents a child’s deviation from the mean log-odds trajectory of behavior *r* for children in class *k*. Within each class we allow for correlation between $u_{ik}^{\left( 1 \right)}$ and $u_{ik}^{\left( 2 \right)}$; for example, a positive correlation would indicate that children with a high (low) probability of disengagement also tend to have a high (low) probability of negative behavior. The model was estimated using the Mplus software (Muthén & Muthén, 1998-2010).

We use the adjusted BIC and LMR statistical criteria to help identify the correct number of classes. The adjusted BIC measures the goodness of fit of the fitted model penalized for model complexity (i.e. number of model parameters and sample size) while the LMR is a modified version of a standard likelihood ratio test (LRT). Improved model fit is demonstrated by lower values on the BIC. The LMR test compares the K-class model to K – 1 class model. Low *p*-values (*p* < 0.05) suggest that the K-class model is favored over the K – 1 class model.

Having established the preferred model as a K-class model, we further assess the fit of the model by examining the precision with which children might be classified into distinct classes. The GMM assigns each child a probability of belonging to each class (posterior class membership probabilities). Conceptually, a child will be assigned a high probability of belonging to a specific class if their temporal behavior sequence is similar to the temporal behavioral sequence of that class. Thus, to investigate the precision with which children might be classified into classes, we assign children to the classes to which they have the highest probabilities (their most likely classes) and then, for each class, we calculate the mean of these assignment probabilities. The higher these means, the more precisely children can be classified into classes.

The coefficients, $\beta_{1k}^{\left( r \right)}$ and $\beta_{2k}^{\left( r \right)}$, are the effects of time and time-squared on the log-odds of disengagement (*r* = 1) versus positivity and negativity (*r* = 2) versus positivity. The estimated coefficients for the quadratic function in each class are shown for the selected 2-class model for younger siblings (Table A1) and the 4-class-model for older siblings (Table A2). However, these are difficult to interpret because the sign of coefficients may not reflect the direction of the effect of time on either of the response probabilities being compared (Retherford & Choe, 1993, p.153). We therefore compute predicted response probabilities from the fitted model to examine the magnitude of the nonlinear time effects on the three types of behavior in each class. The probabilities of disengagement, negativity, and positivity for each class *k* are computed from (1), averaging across simulated draws from the bivariate normal random effects distribution to obtain population-averaged probabilities.

**3. Additional details on analysis of predictors of behavior trajectories**

We examine predictors of class membership by fitting a second multinomial logit model. Specifically, we first allocate children to their most likely latent classes based on the posterior probabilities produced by the GMM. We then estimate a multinomial logit model to the classes to which the children are assigned. To take account of uncertainty in the allocation of children to classes, we use the pseudo-class method proposed by Wang, et al*.* (2005) and implemented in Mplus (Asparouhouv & Muthén, 2007).

**References**

Muthén, L. K. & Muthén, B. O. (1998-2010). *Mplus User’s Guide* (Sixth ed.). Los Angeles, CA: Muthén & Muthén.

Retherford, R. D., & Choe, M. K. (1993). *Statistical Models for Causal Analysis*. New York: Wiley.

| Table A1  *Effects of Time on Log-odds of Disengagement and Negativity (vs. positivity) for Younger Siblings in the Two Latent Classes* | | | | | | |
| --- | --- | --- | --- | --- | --- | --- |
| Parameter | Disengagement vs. positivity | | | Negativity vs. positivity | | |
|  | Estimate | Std. Err. | *p*-value | Estimate | Std. Err. | *p*-value |
| Non-reactive class |  |  |  |  |  |  |
| Intercept | -2.097 | 0.298 | <0.001 | -1.122 | 0.233 | <0.001 |
| Time | 0.084 | 0.050 | 0.092 | -0.018 | 0.038 | 0.637 |
| Time^2^ | -0.003 | 0.002 | 0.050 | 0.001 | 0.001 | 0.385 |
|  |  |  |  |  |  |  |
| Deteriorating class |  |  |  |  |  |  |
| Intercept | -4.690 | 0.627 | <0.001 | -0.798 | 0.224 | <0.001 |
| Time | 0.178 | 0.103 | 0.084 | 0.007 | 0.043 | 0.873 |
| Time^2^ | -0.001 | 0.004 | 0.716 | -0.002 | 0.001 | 0.099 |

| Table A2  *Effects of Time on Log-odds of Disengagement and Negativity (vs. positivity) for Older Siblings in the Four Latent Classes.* | | | | | | |
| --- | --- | --- | --- | --- | --- | --- |
| Parameter | Disengagement vs. positivity | | | Negativity vs. positivity | | |
|  | Estimate | Std. Err. | *p*-value | Estimate | Std. Err. | *p*-value |
| Recovery (1) |  |  |  |  |  |  |
| Intercept | -3.158 | 0.720 | <0.001 | -2.664 | 0.580 | <0.001 |
| Time | -0.120 | 0.081 | 0.137 | 0.345 | 0.097 | <0.001 |
| Time^2^ | 0.007 | 0.002 | 0.005 | -0.016 | 0.004 | <0.001 |
|  |  |  |  |  |  |  |
| Non-reactive (2) |  |  |  |  |  |  |
| Intercept | -2.505 | 0.608 | <0.001 | -0.161 | 0.327 | 0.624 |
| Time | 0.032 | 0.101 | 0.753 | -0.188 | 0.054 | <0.001 |
| Time^2^ | -0.003 | 0.003 | 0.375 | 0.003 | 0.002 | 0.076 |
|  |  |  |  |  |  |  |
| Early disengagement (3) |  |  |  |  |  |  |
| Intercept | -12.416 | 1.900 | <0.001 | -0.252 | 0.308 | 0.413 |
| Time | 1.002 | 0.177 | <0.001 | -0.048 | 0.057 | 0.395 |
| Time^2^ | -0.023 | 0.005 | <0.001 | 0.000 | 0.003 | 0.965 |
|  |  |  |  |  |  |  |
| Deterioration (4) |  |  |  |  |  |  |
| Intercept | -3.194 | 0.545 | <0.001 | -1.474 | 0.427 | 0.001 |
| Time | 0.151 | 0.060 | 0.012 | -0.046 | 0.045 | 0.305 |
| Time^2^ | -0.004 | 0.002 | 0.079 | 0.003 | 0.001 | 0.062 |
